# Thermal decomposition of Zn[(C_6_H_5_)_2_PSSe]_2_ single-source precursor for the chemical vapour deposition of binary and ternary zinc chalcogenides: a theoretical study

**DOI:** 10.1186/s40064-015-1020-9

**Published:** 2015-06-17

**Authors:** Francis Opoku, Noah Kyame Asare-Donkor, Anthony Apeke Adimado

**Affiliations:** Department of Chemistry, Kwame Nkrumah University of Science and Technology, Kumasi, Ghana

**Keywords:** Zinc, Thermodynamic, Mechanistic, Density functional study, Gas phase

## Abstract

The mechanistic pathways for the formation of zinc selenide, zinc sulphide and ternary zinc selenide sulphide in the gas phase decomposition of Zn[(C_6_H_5_)_2_PSSe]_2_ single-source precursor has been explored on the singlet and doublet potential energy surfaces using density functional theory method in order to understand the thermodynamic and kinetic properties. The optimized geometries and the predicted energies were obtained using the M06 functional and a combination of the basis sets LACVP* for Zn and 6-31(d) for light atoms. The rate constants of each elementary reaction have been calculated using the transition state theory. The results indicate that the steps that lead to ternary ZnSe_x_S_1−x_ formation on both the singlet and doublet potential energy surfaces is favoured kinetically and thermodynamically over those that lead to ZnSe and ZnS formation. Density functional theory calculations of the gas phase decomposition of the complex indicate that the deposition of ternary ZnSe_x_S_1−x_ in chemical vapour deposition may involve more than one step but the steps that lead to its formation are consistent with a dominant role for both thermodynamic and kinetic factors. The theoretical study showed important insights as a general tool to anticipate the gas phase decomposition mechanism of a novel precursor when direct experimental measurements are not available.

## Background

Transition metal chalcogenides have been of considerable technological applications such as solar energy conversion, solar control coatings, microelectronic devices, catalysts, sensors, optical filters and laser sources (Yamaguchi et al. 1996; Teteris 2003; Savadogo 1998; Sang et al. 2002). Structural information such as geometrical and electronic configurations, molecular dynamics, and thermodynamic and magnetic properties of the dichalcogenophosphinato complexes is important to understand the different factors influencing their practically useful properties (Artem’ev et al. 2014).

The mixed crystals of II–VI compound semiconductors have attracted much attention for applications in optical devices (El Haj Hassan et al. 2007). Indeed, the easiest way to change artificially the electronic and optical properties of semiconductors is by forming their alloys (Gunshor and Nurmikko 1997). It seems therefore very interesting to study ZnS and ZnSe mixed in the ZnS_x_Se_1−x_ ternary alloys.

An experimental observation of labile and reactive intermediates on the surface is ultimately challenging and difficult, since the experiments have great difficulties in measuring the intermediates and the transition states in the high-temperature system with complicated reactions. Optimising the CVD conditions such that higher-purity materials are obtained at a higher growth rate requires knowledge of the deposition chemistry obtained by performing specially designed experiments and/or modelling and simulation (Opoku et al. 2014). Due to the difficulty of assessing such a reaction mechanism by experiment, theoretical calculations can be an excellent means of exploring these processes on a molecular scale. Knowledge of thermodynamic and kinetic parameters obtained from density function theory calculation is important to understand and optimise deposition conditions require for selective growth process in chemical vapour deposition. Therefore, understanding the kinetics of gas phase decomposition reactions of single source precursor is becoming more and more important. The good correspondence between DFT results and experimental data has led to DFT playing a pivotal role in the prediction of the reaction rates of complex species important for gas-phase reactions of single-source precursors (Hohenberg and Kohn 1964).

**Figure 1:**
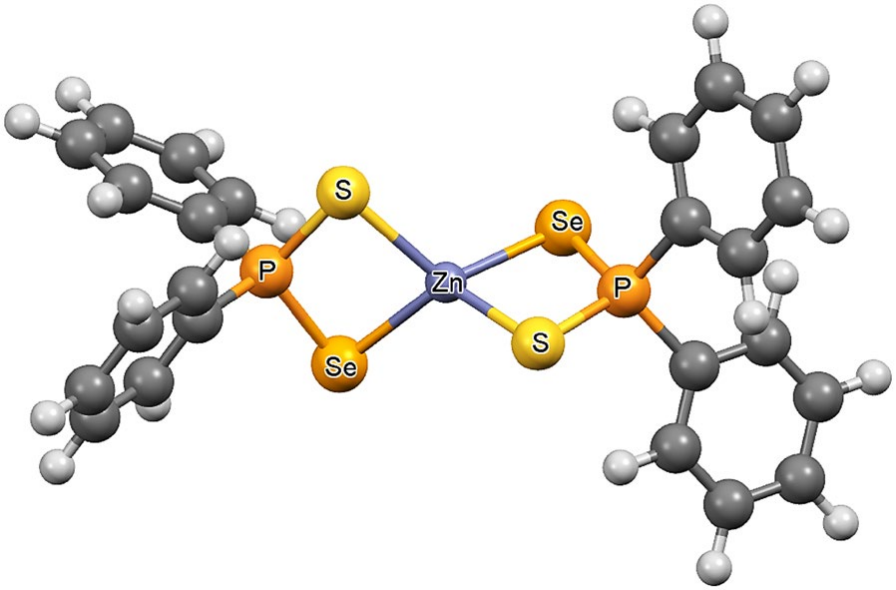
Optimised structure of Zn[(C_6_H_5_)_2_PSSe]_2_ single-source precursor.

Data on the structural chemistry of the Zn[(C_6_H_5_)_2_PSSe]_2_ precursor are scarce. In particular, experimental investigations and quantum-chemical models of the Zn[(C_6_H_5_)_2_PSSe]_2_ precursor have not been reported. Opoku, Asare-Donkor and Adimado had studied the mechanisms of the gas phase decomposition of Cd[(^*i*^Pr)_2_PSSe]_2_, Pb[(C_6_H_5_)_2_PSSe]_2_ and Zn[(^*i*^Pr)_2_PSSe]_2_ single source precursors (Opoku et al. 2014; 2015a, b). In continuation of such efforts, we have analysed the decomposition behaviour of the zinc (II) thioselenophosphinate, Zn[(C_6_H_5_)_2_PSSe]_2_ in the gas phase. A mechanism that consists of 24 reactions has been proposed to account for the gas-phase decomposition of the Zn[(C_6_H_5_)_2_PSSe]_2_ precursor. In this study we focus: (1) on theoretical studies performed to understand the reaction mechanisms of the Zn[(C_6_H_5_)_2_PSSe]_2_ single-source precursor; (2) on computational studies performed to assess the performance of the precursor depending on the ligand employed. Therefore, the aim of this study is to show how the theoretical studies can be a crucial help to understand and predict reaction mechanisms, providing a detailed picture at atomistic level of the intermediates involved in the decomposition and unveiling the electronic and structural properties of the precursor (Figure 1).

## Computational details

All the calculations were done using the M06 hybrid density functional. The M06 is a novel hybrid *meta* functional with good accuracy and has been parameterized for modelling organometallic and inorganometallic thermochemistry, non-covalent interactions and kinetics for systems containing transition metal elements (Zhao and Truhlar 2008; Zhao et al. 2014). Open shell systems were treated using unrestricted density functional theory. Geometry optimizations were performed using a standard valence LACVP* basis set as implemented in the Spartan Molecular Modelling program (Wave function 2010). For the first- and second-row elements, LACVP implies a 6-31G double-ξ basis set. For the zinc atoms, LACVP uses a nonrelativistic effective core potential (LACVP* uses the 6-31G* basis set for all light elements and the Hay-Wadt ECP and basis set for Zn; see: Hay and Wadt 1985a, b; Wadt and Hay 1985), where the valence part is essentially of double-ξ quality. The starting geometries of the molecular systems were constructed using Spartan’s graphical model builder and minimized interactively using the sybyl force field (Clark et al. 1989). Local minima were optimized using the Spartan ‘10 v1.1.0 Molecular Modelling program (Wave function 2010). A normal mode of analysis was performed to verify the nature of the stationary point and equilibrium geometries were characterized by the absence of imaginary frequencies. The transition state structures were located by series of constrained geometry optimization in which the breaking bonds were fixed at various lengths and optimized the remaining internal coordinates. The approximate stationary points located from such a procedure were then fully optimized using the standard transition state optimization procedure in Spartan (Aniagyei et al. 2013). All first-order saddle points were shown to have an imaginary vibrational frequency along the reaction coordinate.

The rate constants were computed using the transition state theory for the selected reaction pathways (Benson 1960; Glasstone et al. 1941) and assuming that the transmission coefficient, *κ* is equal to 1.

1 $${\text{k}}_{\text{uni}} = \left( {\frac{{\kappa {\text{k}}_{\text{B}} {\text{T}}}}{\text{h}}} \right)\exp \left( {\frac{{\Delta {\text{G}}^{\ddag } }}{\text{RT}}} \right)$$

2 $${\text{K}}_{\text{eq}} = { \exp }({-}\Delta {\text{G}}^{\text{o}} /{\text{RT}})$$

The recombination rate constants (k_rec_) from the kinetics is

3 $${\text{k}}_{\text{rec}} = {\text{ K}}_{\text{eq}} \times {\text{k}}_{\text{uni}}$$

where ΔG^‡^ is the Gibbs free activation energy, ΔG^o^ is the Gibbs free energy, and k_B_ and h are the Boltzmann and Planck constants, respectively.

## Mechanistic considerations

**Scheme 1:**
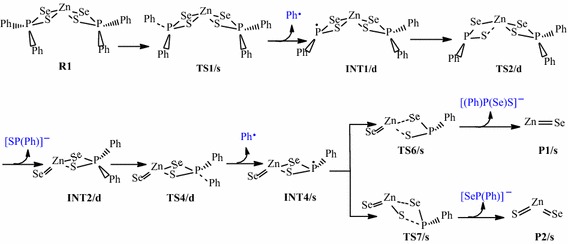
Proposed decomposition pathway of (C_6_H_5_)P(Se)S–Zn–Se intermediate.

**Scheme 2:**
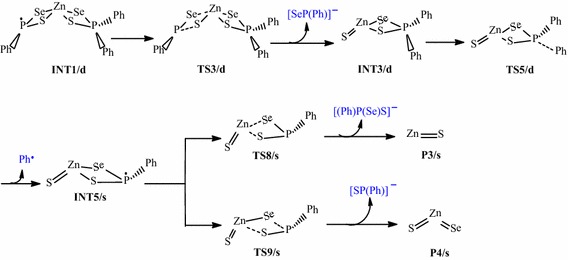
Proposed decomposition pathway of (C_6_H_5_)P(Se)S–Zn–S intermediate.

**Scheme 3:**
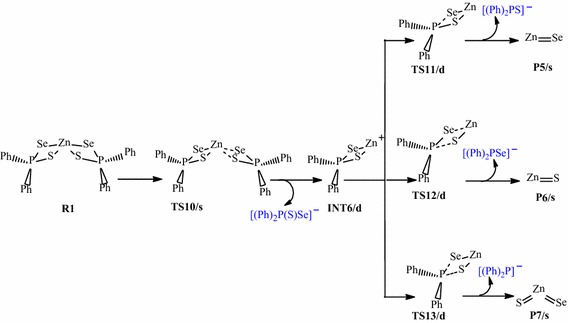
Proposed decomposition pathway of (C_6_H_5_)_2_P(Se)S–Zn intermediate.

**Scheme 4:**
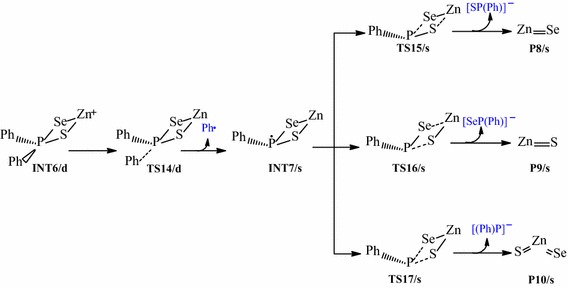
Proposed decomposition pathway of (C_6_H_5_)P(Se)S–Zn intermediate.

The reaction pathways for the gas phase decomposition of Zn[(C_6_H_5_)_2_PSSe]_2_ precursor were based on the schemes suggested by Opoku et al. (2014, 2015a, b) and Akhtar et al. (2011). The species considered are (1) the dissociation of ZnSe, (2) the dissociation of ZnS, and (3) the heterogeneous P–S and P–Se bond cleavages to form a ternary ZnSe_x_S_1−x_. These have been presented in Schemes 1, 2, 3 and 4. The activation and reaction free energies were computed at T = 800K.

## Results and discussion

### Optimized Geometry of Zn[(C_6_H_5_)_2_PSSe]_2_ precursor

Table 1 lists the bond angles and bond lengths of the Zn[(C_6_H_5_)_2_PSSe]_2_ precursor. The geometry at the zinc atom is distorted tetrahedral. The Zn–Se bond lengths, 2.50 Å, are slightly longer than the Zn–S distance, 2.43 Å. The S–Zn–Se angle (89.50° and 89.53°) is smaller than the S–P–Se angle (108.5°) due to the large amount of repulsion between the lone pairs of electrons of phosphorus with those of zinc. The wider S–Zn–Se bond angle of 120.5° was as a result of the proximity of the non-coordinating S- and Se-donor atoms to the Zn(II) atom. The geometrical parameters are in reasonable agreement with theoretically determined data on Zn[(^*i*^Pr)_2_PSSe]_2_ precursor (Opoku et al. 2015b).

**Table 1:** Comparison of the calculated geometries of Zn[(C_6_H_5_)_2_PSSe]_2_ and Zn[(^*i*^Pr)_2_PSSe]_2_ precursor at the M06/LACVP* level of theory (bond lengths in angstroms and bond angles in degrees)

Bond lengths	M06/LACVP*		Bond angles	M06/LACVP*	
P_1_–S_1_	2.05	2.13^a^	S_1_–P_1_–Se_1_	108.5	97.97^a^
P_1_–Se_1_	2.22	2.24^a^	S_2_–Zn–Se_2_	89.5	87.46^a^
S_2_–P_2_	2.05	2.14^a^	Se_1_–Zn–S_1_	89.5	87.85^a^
Se_2_–P_2_	2.22	2.24^a^	S_1_–Zn–S_2_	118.2	52.56^a^
Zn–S_2_	2.43	2.20^a^	Se_1_–Zn–Se_2_	121.6	150.06^a^
Zn–Se_1_	2.50	2.54^a^	S_1_–Zn–Se_2_	120.5	120.53^a^
S_1_–Zn	2.42	2.20^a^	Se_1_–Zn–S_2_	120.7	120.02^a^
Se_2_–Zn	2.50	2.55^a^	Se_2_–P_2_–S_2_	108.5	97.44^a^

^a^Data from Opoku et al. (2015b).

### Overall decomposition of Zn[(C_6_H_5_)_2_PSSe]_2_ precursor

**Figure 2:**
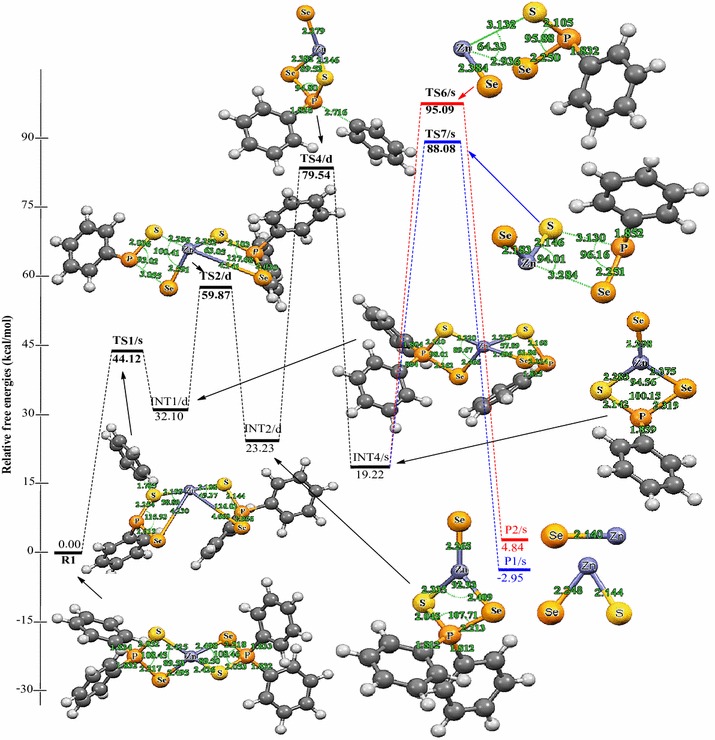
Energy profile of the decomposition pathway of (C_6_H_5_)PSSe–Zn–Se intermediate. Data in the path are the relative Gibbs free energies (in kcal/mol and bond distances in Å) obtained at M06/LACVP* level of theory.

**Figure 3:**
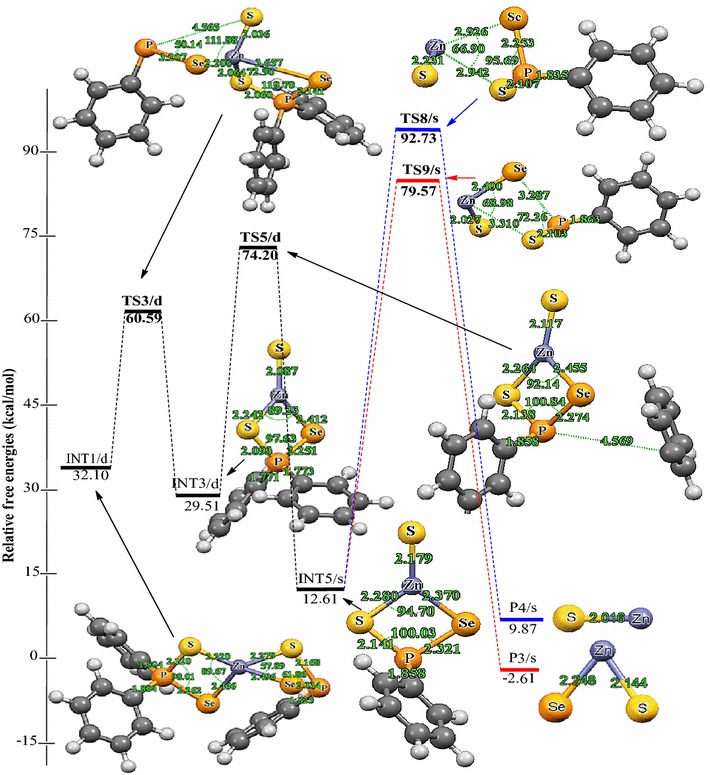
Energy profile of the decomposition pathway of (C_6_H_5_)PSSe–Zn–S intermediate. Data in the path are the relative Gibbs free energies (in kcal/mol and bond distances in Å) obtained at M06/LACVP* level of theory.

Relative energies for all species postulated to be involved in the reaction mechanism of the decomposition of Zn[(C_6_H_5_)_2_PSSe]_2_ precursor in Schemes 1 and 2 are depicted in Figures 2 and 3. The geometries of all molecular structures taking part in the reactions under investigation were fully and independently optimized using analytical gradients at the M06 levels with the LACVP* basis set. At each level of theory the potential energy surface was explored independently for the possible existence of transition states and intermediate complexes. The calculated lengths of the dissociation of Zn–Se and Zn–S bonds are 2.94 and 3.13 Å for TS6/s and, 2.93 and 2.94 Å for TS8/s. On the other hand, the calculated lengths of Zn–Se and P–S bonds are 3.28 and 3.13 Å, respectively for TS7/s. In TS9/s the dissociation of Zn–S and P–Se bonds are 3.31 and 3.29 Å. All the complexes retain symmetry of the C_1_ point group.

The calculated Gibbs free energy of activation and reaction energy necessary for the formation of the (C_6_H_5_)_2_PSSe–Zn–SeSP(C_6_H_5_) intermediate on the double potential energy surface through a singlet transition state TS1/s are +44.12 and +32.10 kcal/mol, respectively. The consequent decomposition of the (C_6_H_5_)_2_PSSe–Zn–SeSP(C_6_H_5_) intermediate through singlet transition state TS2/d requires a barrier of +27.77 kcal/mol (Figure 2) and a reaction energy of −8.80 kcal/mol to form the (C_6_H_5_)_2_PSSe–Zn–Se intermediate. It is possible for the (C_6_H_5_)_2_PSSe–Zn–Se intermediate to decompose in two ways. On the singlet surface, the decomposition of the (C_6_H_5_)_2_PSSe–Zn–SeSP(C_6_H_5_) intermediate to form the (C_6_H_5_)_2_PSSe–Zn–S intermediate has an activation barrier of +28.49 kcal/mol and reaction energy of −2.59 kcal/mol. The formation of the singlet (C_6_H_5_)_2_PSSe–Zn–Se intermediate by the dissociation of a phenyl radical from the (C_6_H_5_)PSSe–Zn–Se intermediate through the doublet transition state requires an activation barrier of +56.24 kcal/mol and reaction energy of −4.01 kcal/mol. A singlet (C_6_H_5_)PSSe–Zn–S intermediate has been found to be 12.89 kcal/mol more stable than the C_6_H_5_)PSSe–Zn–Se intermediate.

The subsequent decomposition of the (C_6_H_5_)PSSe–Zn–Se intermediate through a singlet transition state to form ZnSe + [(C_6_H_5_)PSeS]^−^ has an activation barrier of +68.86 kcal/mol (Figure 2) while the decomposition of the (C_6_H_5_)PSSe–Zn–Se intermediate through a singlet transition state to form ternary ZnSe_x_S_1−x_ + [(C_6_H_5_)PSe]^−^ has a barrier of +75.87 kcal/mol. In a related study on the gas phase decomposition of Zn[(^*i*^Pr)_2_PSSe]_2_ precursor, Opoku et al. (2015b) found the activation barrier for the formation of ZnSe and ternary ZnSe_x_S_1−x_ to be +56.22 and +67.34 kcal/mol (Table 2).

**Table 2:** Calculated activation barriers and reaction energy of the last step of the various reactions of the Zn[(C_6_H_5_)PSSe]_2_ and Zn[(^*i*^Pr)_2_PSSe]_2_^b^ complexes

Reaction pathway	Activation barrier		Reaction energy	
INT4/s → P1/s	+68.86	+56.22^a^	−14.38	−31.05^a^
INT4/s → P2/s	+77.87	+67.34^a^	−22.17	−46.80^a^
INT5/s → P3/s	+80.12	+49.22^a^	−2.74	−22.64^a^
INT5/s → P4/s	+66.96	+53.65^a^	−15.22	−46.47^a^
INT6/d → P5/s	+17.71	+0.82^a^	−47.89	−28.57^a^
INT6/d → P6/s	+22.18	+5.35^a^	−40.74	−23.50^a^
INT6/d → P7/s	+12.09	+58.86^a^	−58.63	−63.55^a^
INT7/s → P8/s	+40.47	+12.64^a^	−26.42	−16.16^a^
INT7/s → P9/s	+38.54	+20.11^a^	−21.66	−9.50^a^
INT7/s → P10/s	+24.32	+21.99^a^	−33.15	−39.01^a^

^a^Data from Opoku et al. (2015b).

Deposition rates from the phenyl phosphinato complex, Zn[(C_6_H_5_)_2_PSSe]_2_ have been observed to be lower than those from the isopropyl phosphinato complex. These differences are attributed to the higher dissociation energy of the P–C bond in phenyl phosphinato complex and also greater electron withdrawing nature of the phenyl substituent, as compared to isopropyl. The higher activation energy for phenyl phosphinato is consistent with cleavage of the stronger phosphinato P–C bond before or during the rate-determining step of the deposition process (Table 2).

Thus, the overall barrier for the decomposition of the (C_6_H_5_)PSSe–Zn–Se intermediate to give the ternary ZnSe_x_S_1−x_ is higher than the activation barrier for the ZnSe formation pathway. The reaction which would lead back to the reactant is energetically less favourable due to the larger barrier. The ternary ZnSe_x_S_1−x_ dissociation pathway is only 7.79 kcal/mol more stable than the ZnSe dissociation pathway. Moreover, it is possible for the (C_6_H_5_)PSSe–Zn–S intermediate to decompose in two ways. The formation of the singlet ZnS + [(C_6_H_5_)PSeS]^−^ through a singlet transition state has an activation barrier of +80.12 kcal/mol and a reaction energy of −2.74 kcal/mol. The formation of singlet ternary ZnSe_x_S_1−x_ + [(C_6_H_5_)PS]^−^ through the singlet transition state by the dissociation of the Zn–S_2_ and Zn–Se_1_ bonds from the (C_6_H_5_)PSSe–Zn–S intermediate has an activation barrier of +66.96 kcal/mol and reaction energy of −15.22 kcal/mol.

In study of the decomposition of Zn[(^*i*^Pr)_2_PSSe]_2_ precursor, Opoku et al. (2015b) found the ZnS dissociation pathway to be the most favourable pathway. However, in this work the dissociation of ternary ZnSe_x_S_1–x_ is kinetically more favourable than the ZnSe and ZnS dissociation pathways but the reaction is slow by the highest activation barrier. The barrier along this pathway is +8.91 and +13.16 kcal/mol lower than the ZnSe and ZnS dissociation pathway, respectively. Ternary ZnSe_x_S_1−x_ formed from the optimization of the (C_6_H_5_)PSSe–Zn–S intermediate is the most stable species on the reaction PES; the corresponding unimolecular and equilibrium rate constants are 3.05 × 10^−9^/s and 8.19 × 10^1^ cm^3^/mol, respectively (Table 3).

**Table 3:** Calculated rate constants for the gas phase decomposition of Zn[(C_6_H_5_)PSSe]_2_ at 800K

Reaction pathway	k_uni_ (s^−1^)	K_eq_	k_rec_ (s^−1^)
INT4/s → P1/s	1.76 × 10^−13^	2.83 × 10^−4^	4.97 × 10^−17^
INT4/s → P2/s	7.75 × 10^−13^	5.82 × 10^−8^	4.51 × 10^−20^
INT5/s → P3/s	1.44 × 10^−11^	1.45 × 10^2^	2.10 × 10^−9^
INT5/s → P4/s	3.05 × 10^−9^	8.19 × 10^1^	2.50 × 10^−7^
INT6/d → P5/s	9.34 × 10^−3^	1.45 × 10^7^	1.36 × 10^5^
INT6/d → P6/s	5.61 × 10^−4^	8.33 × 10^1^	4.67 × 10^−2^
INT6/d → P7/s	3.20 × 10^−1^	1.08 × 10^15^	3.47 × 10^14^
INT7/s → P8/s	5.87 × 10^−8^	1.41 × 10^−6^	8.30 × 10^−14^
INT7/s → P9/s	1.98 × 10^−7^	4.58 × 10^−10^	9.05 × 10^−17^
INT7/s → P10/s	1.52 × 10^−3^	1.21 × 10^−1^	1.84 × 10^−4^

**Figure 4:**
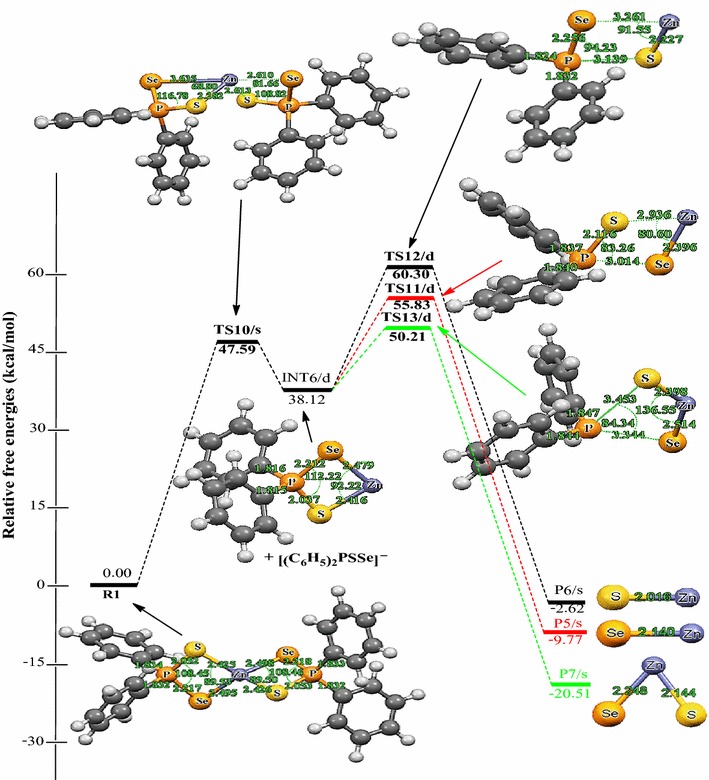
Energy profile of the decomposition pathway of (C_6_H_5_)_2_P(Se)S–Zn intermediate. Data in the path are the relative Gibbs free energies (in kcal/mol and bond distances in Å) obtained at M06/LACVP* level of theory.

The initial decomposition of Zn[(C_6_H_5_)_2_PSSe]_2_ precursor was further explored on the doublet PES (i.e. TS-[10-13] in Scheme 3). The energy profile of the gas phase decomposition of the (C_6_H_5_)_2_PSSe–Zn intermediate and the optimized structures involved in the reaction mechanisms are shown in Figure 4. Density functional theory (DFT) optimisation shows that Zn[(C_6_H_5_)_2_PSSe]_2_ can have a singlet ground-state electronic structure for the products and a doublet electronic ground-state structure for the intermediate, (C_6_H_5_)_2_PSSe–Zn. The Zn–S and P–Se bonds increases from 2.42 and 2.21 Å in the reactant (INT6/d) to 2.93 and 3.01 Å in the transition state (TS11/d) while the Zn–Se and P–S bonds increases from 2.48 and 2.04 Å in the reactant to 3.26 and 3.14 Å in the transition state (TS12/d). Also in TS13/d optimised structure, the P–Se and P–S bonds increases from 2.21 and 2.04 Å in the reactant to 3.34 and 3.45 Å in the transition state.

The decomposition of the Zn[(C_6_H_5_)_2_PSSe]_2_ to give the doublet (C_6_H_5_)_2_PSSe–Zn intermediate through the singlet transition state has an activation barrier and reaction energy of +47.29 and +38.12 kcal/mol, respectively. The decomposition of the doublet four-membered (C_6_H_5_)_2_PSSe–Zn intermediate to form ZnSe + [(C_6_H_5_)_2_PS]^−^ has an activation barrier of +17.71 kcal/mol and a reaction energy of −47.89 kcal/mol while the decomposition of the doublet four-membered (C_6_H_5_)_2_PSSe–Zn intermediate to form ZnS + [(C_6_H_5_)_2_PSe]^−^ has an activation barrier of +22.18 kcal/mol and a reaction energy of −40.74 kcal/mol. The formation of ZnSe through the dissociation of Zn–S and P–Se bonds from (^*i*^Pr)_2_PSSe–Zn intermediate (TS11/s in Scheme 3) which was reported in the work of Opoku et al. (2015b) has been found to have a barrier and reaction energy of +0.82 and −28.57 kcal/mol, respectively on the singlet surface.

Also, the formation of the ternary ZnSe_x_S_1−x_ + [(C_6_H_5_)_2_P]^−^ through direct dissociation of the P–Se_1_ and P_1_–S_1_ from the (C_6_H_5_)_2_PSSe–Zn intermediate has an activation barrier of +12.09 kcal/mol and a reaction energy of −58.63 kcal/mol. The barrier along this route is only 5.62 kcal/mol lower than the barrier for the ZnSe formation pathway. The activation barriers for the formation of the singlet ZnSe and ZnS (17.71 and 22.18 kcal/mol) are higher than the activation barrier for the formation of ternary ZnSe_x_S_1−x_. Moreover, this decomposition pathway has the lowest activation free-energy barrier and will proceed very rapidly; the corresponding unimolecular and recombination rate constant are 3.20 × 10^−1^/s and 3.47 × 10^14^ cm^3^/mol/s, respectively, see Table 3. Thus overall, the formation of ZnSe and ZnS by the decomposition of the (C_6_H_5_)_2_PSSe–Zn intermediate cannot compete favourably, both kinetically and thermodynamically with the ternary ZnSe_x_S_1−x_ formation pathway.

**Figure 5:**
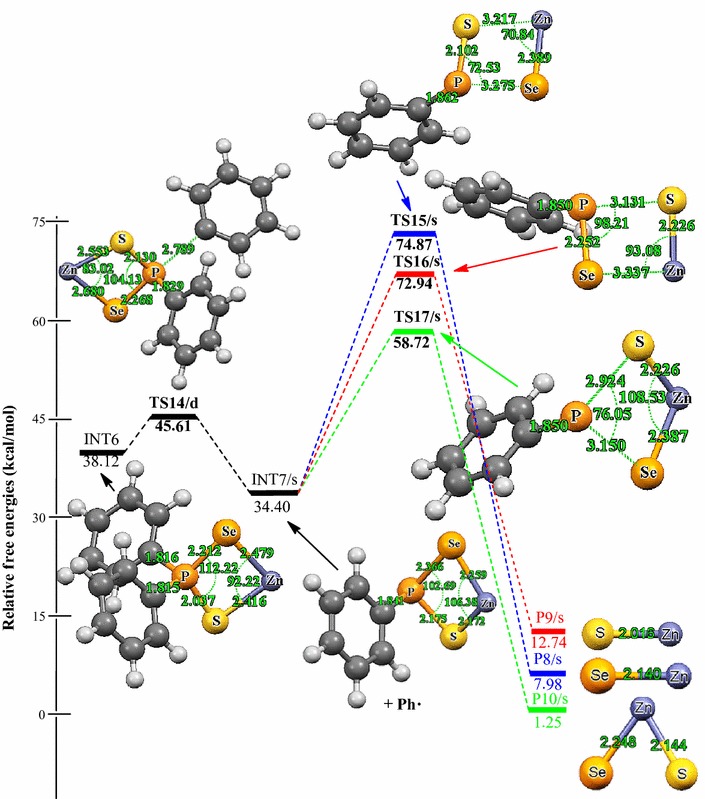
Energy profile of the decomposition pathway of (C_6_H_5_)P(Se)S–Zn intermediate. Data in the path are the relative Gibbs free energies (in kcal/mol and bond distances in Å) obtained at M06/LACVP* level of theory.

The energies as well as the optimized molecular structures obtained for the formation of ZnSe, ZnS and ternary ZnSe_x_S_1−x_ from the (C_6_H_5_)PSSe–Zn intermediate in Scheme 4 are summarized in Figure 5. Density functional theory (DFT) optimisation of the reactant (C_6_H_5_)_2_PSSe–Zn shows that (C_6_H_5_)_2_PSSe–Zn can have a singlet ground-state electronic structure for the products and a doublet electronic ground-state structure for the intermediate, (C_6_H_5_)PSSe–Zn. The optimised geometry of the transition state (TS) structure reveals that the Zn–S bond elongates from 2.17 to 3.22 Å (TS15/s) and the P–Se bond lengthens from 2.36 to 3.28 (TS15/s) and 2.36 to 3.15 Å (TS17/s) while the optimised TS structure reveals that the Zn–Se bond elongates from 2.26 to 3.34 Å (TS16/s) and the P–S bond lengthens from 2.17 to 3.13 (TS16/s) and 2.17 to 2.92 Å (TS17/s). The (C_6_H_5_)PSSe–Zn intermediate is 3.72 kcal/mol exergonic. The singlet transition state TS15/s leading to the formation of ZnSe + [C_6_H_5_PS]^−^ from the (C_6_H_5_)PSSe–Zn intermediate is 7.49 kcal/mol above the reactant. The formation of the ZnS + [C_6_H_5_PSe]^−^ through the singlet transition state TS16/s by the abstraction of Zn–Se_1_ and P_1_–S_1_ bonds from the (C_6_H_5_)PSSe–Zn intermediate is exergonic by 21.66 kcal/mol and has an activation barrier of +38.54 kcal/mol (Figure 5). The dissociation of P_1_–Se_1_ and P_1_–S_2_ bonds from the (C_6_H_5_)PSSe–Zn intermediate through a singlet transition state TS17/s leads to the formation of ternary ZnSe_x_S_1–x_ + [C_6_H_5_P]^−^. This pathway has an activation barrier of +24.32 kcal/mol and the resulting product (P10/s) is −33.15 kcal/mol; 6.73 and 11.49 kcal/mol more stable than the ZnSe and ZnS dissociation pathway, respectively. Figure 5 shows that the dissociation of P_1_−Se_1_ and P_1_–S_2_ bonds from the (C_6_H_5_)PSSe–Zn complex to form the ternary ZnSe_x_S_1−x_ has a lower activation barrier than the ZnSe and ZnS dissociation pathway. A rate constants of 1.52 × 10^−3^/s, 1.21 × 10^−1^ cm^3^/mol and 1.84 × 10^−4^ cm^3^/mol/s were estimated for this process (Table 3).

### Spin density

**Figure 6:**
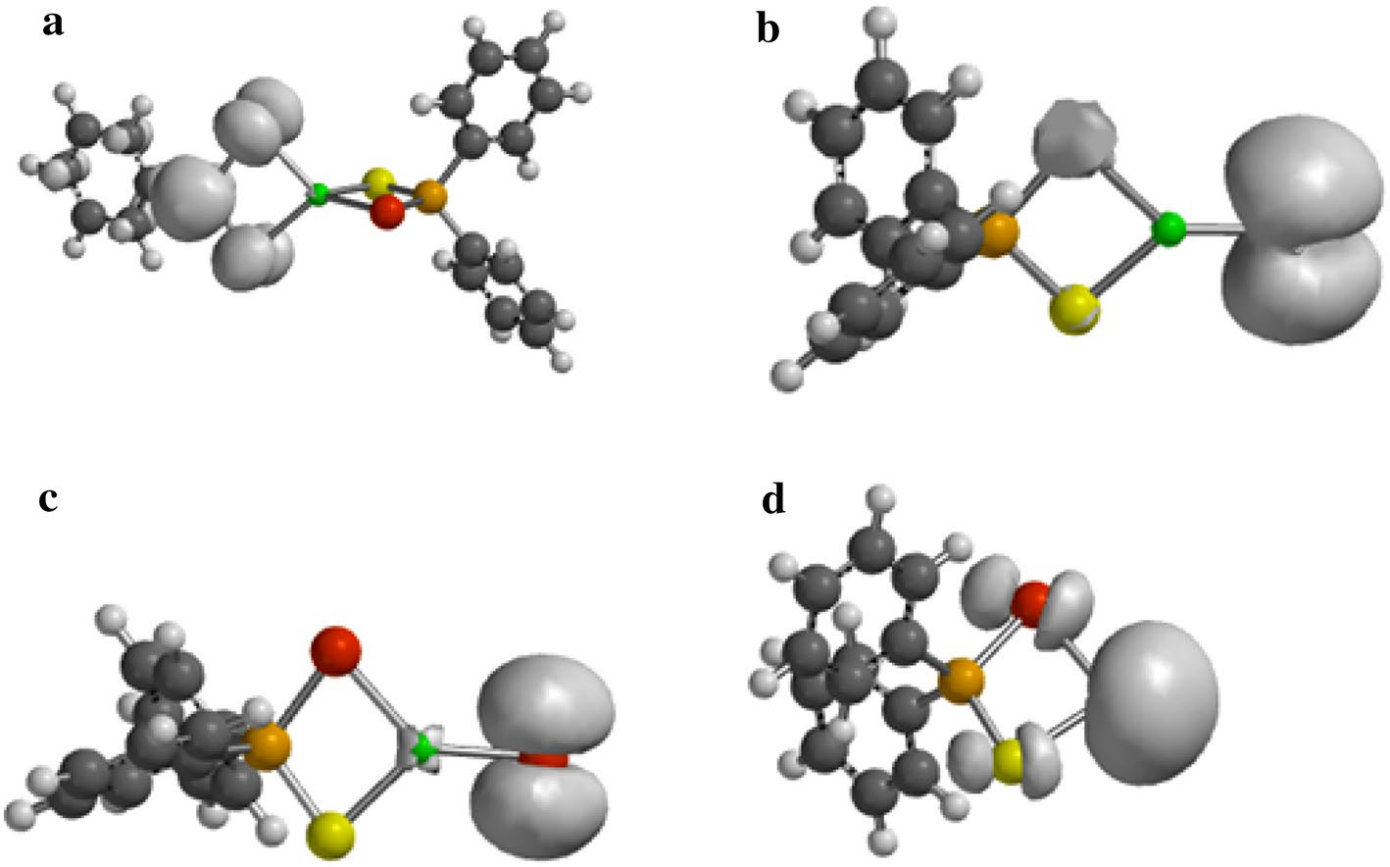
Spin-density distribution for **a** (C_6_H_5_)_2_PSSe–Zn–SeSP(C_6_H_5_), **b** (C_6_H_5_)_2_PSSe–Zn–S, **c** (C_6_H_5_)_2_PSSe–Zn–Se and **d** (C_6_H_5_)_2_PSSe–Zn complexes. Isosurfaces ±0.003 a.u.

The spin density distribution map of the gas phase decomposition of Zn[(C_6_H_5_)_2_PSSe]_2_ single-source precursor and its species has been explored at the same level of theory reported herein. Figure 6a shows a spin density distributed only on one half of the thioselenophosphinate ligand with little/no zinc contribution. The spin density is also symmetrically delocalized on the phenyl group. In Figure 6b the spin density is exclusively distributed on the selenium atom at both side of the ligand. The spin density map in Figure 6c resembles that of a *d*-orbital with a large positive spin density at the sulphur atom. Figure 6d shows a strong zinc contribution with a positive spin density localized on the bridging ligand.

**Figure 7:**
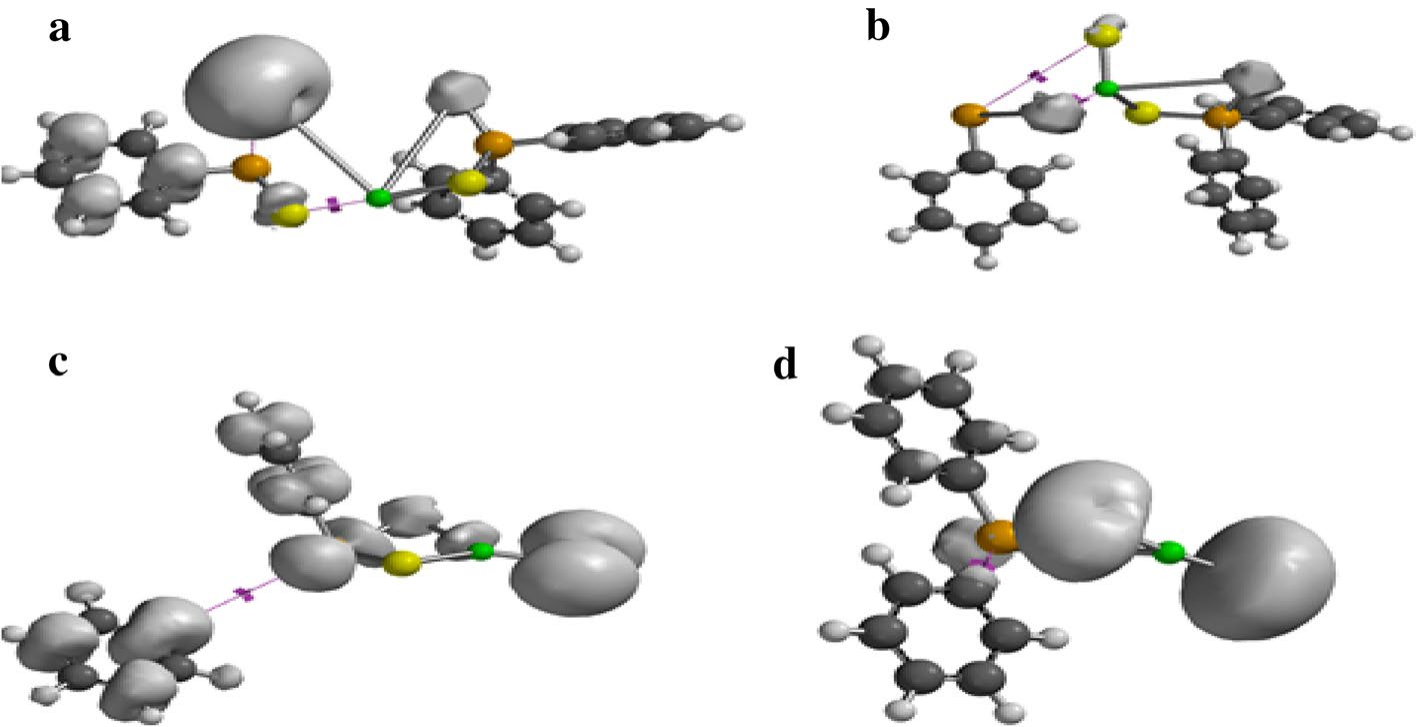
Spin-density distribution for **a** (C_6_H_5_)_2_PSSeZnSe…SP(C_6_H_5_), **b** (C_6_H_5_)_2_PSSeZnS…SeP(C_6_H_5_), **c** C_6_H_5_…(C_6_H_5_)PSSeZnSe and **d** C_6_H_5_…(C_6_H_5_)PSSeZnS complexes. Isosurfaces ±0.003 a.u.

As shown in Figure 7a, d, the spin density is entirely localized on the bridging ligand. Additional spin density is symmetrically delocalized on the phenyl group with little or no metal contribution (Figure 7a, c).

**Figure 8:**
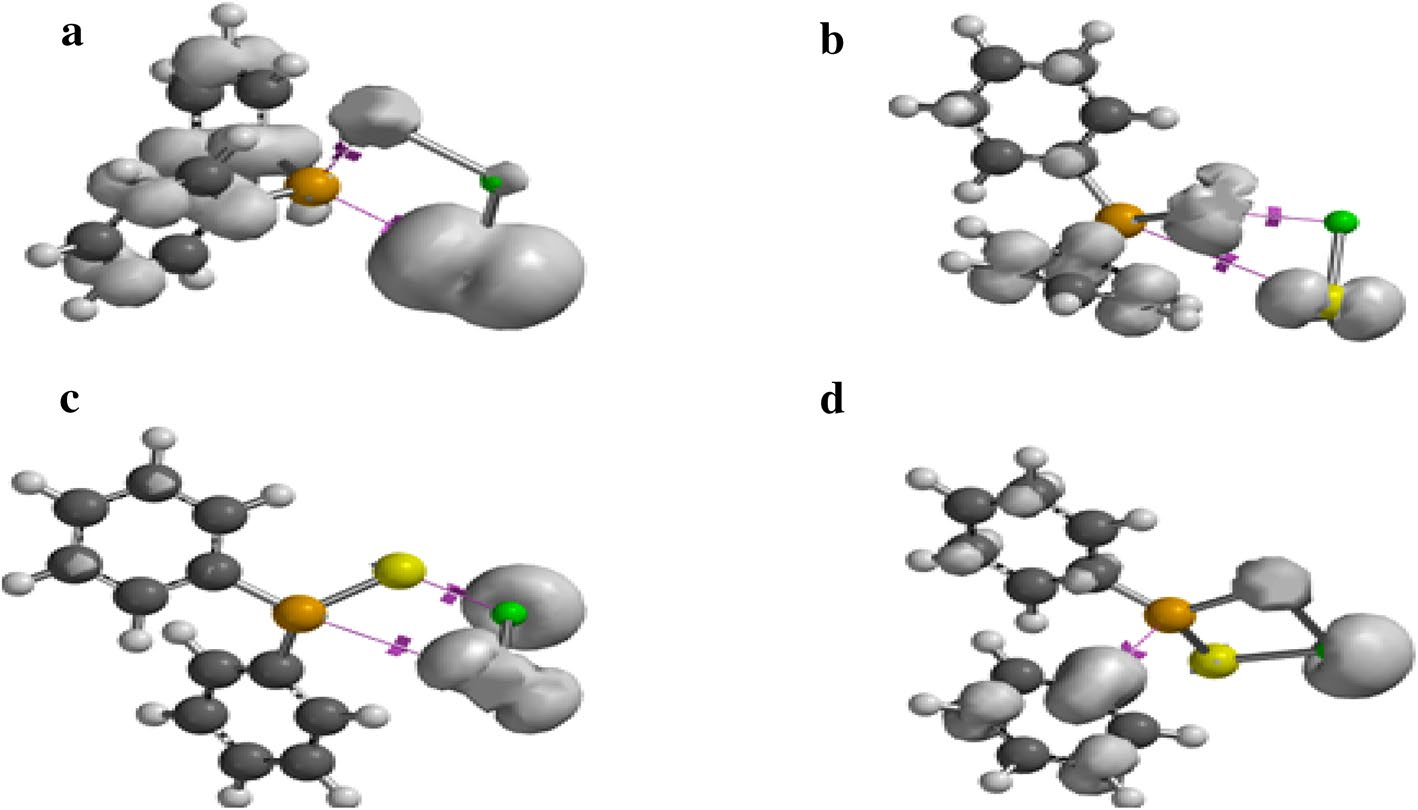
Spin-density distribution for **a** (C_6_H_5_)_2_P…ZnSeS, **b** (C_6_H_5_)_2_PSe…ZnS, **c** (C_6_H_5_)_2_PS…ZnSe and **d** C_6_H_5_…(C_6_H_5_)PSSeZn complex. Isosurfaces ±0.003 a.u.

Figure 8a–d show a positive spin distributed on both the ligand and the metal atom with an exception of (C_6_H_5_)PS.ZnSe complex which shows no metal contribution. Figure 8a, b, d show the same distribution of delocalization of positive spin on the phenyl group.

### Orbital analysis

**Figure 9:**
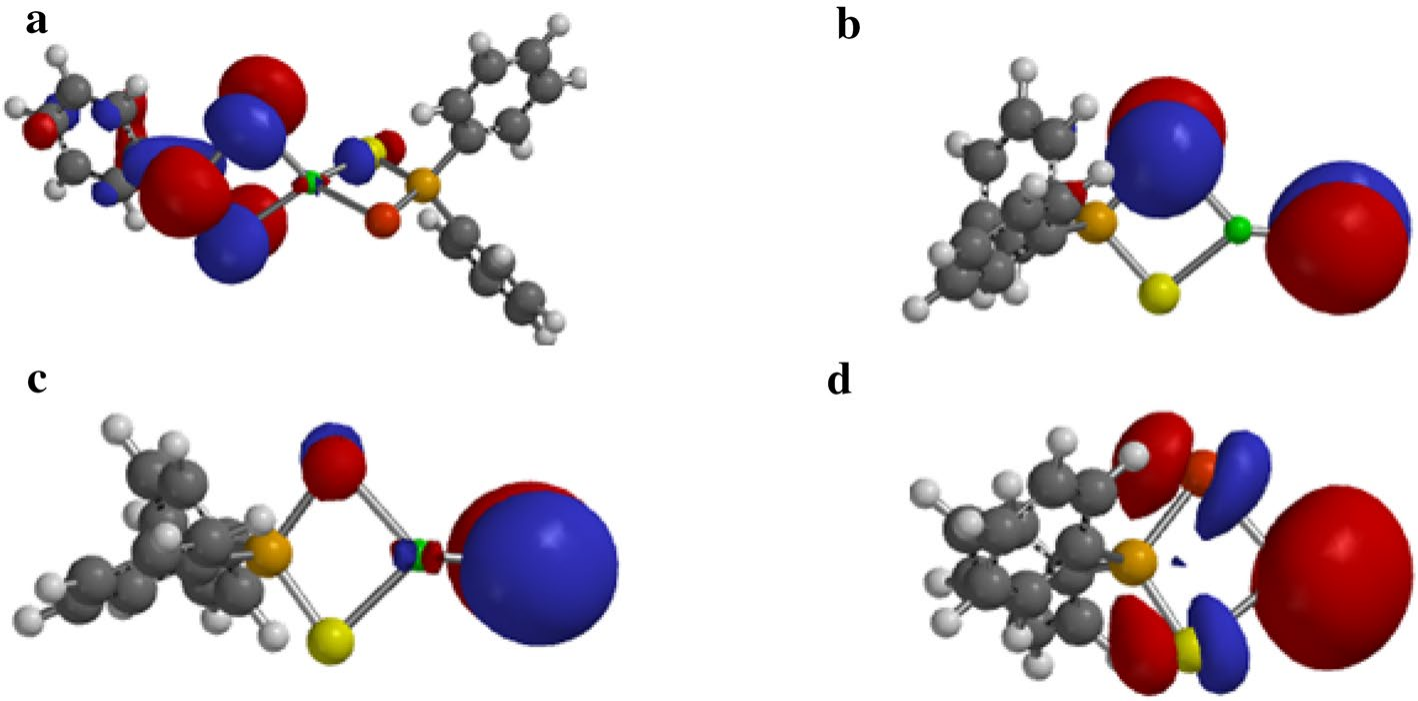
Singly occupied molecular orbitals for **a** (C_6_H_5_)_2_PSSe–Zn–SeSP(C_6_H_5_), **b** (C_6_H_5_)_2_PSSe–Zn–S, **c** (C_6_H_5_)_2_PSSe–Zn–Se and **d** (C_6_H_5_)_2_PSSe–Zn complexes. Isosurfaces ±0.032 a.u.

The single occupied molecular orbital (SOMO) analysis of Zn[(C_6_H_5_)_2_PSSe]_2_ single-source precursor and its species has been explored. In Figure 9a, c the electron density at the metal atom resembles that of dxy-orbital. The SOMO of (C_6_H_5_)_2_P(Se)S–Zn intermediate shows a distribution of electron density on both the metal and the ligand, predominantly on the zinc atom. In Figure 9b, the electron density is entirely distributed on the selenium atom with no metal and sulphur contribution.

**Figure 10:**
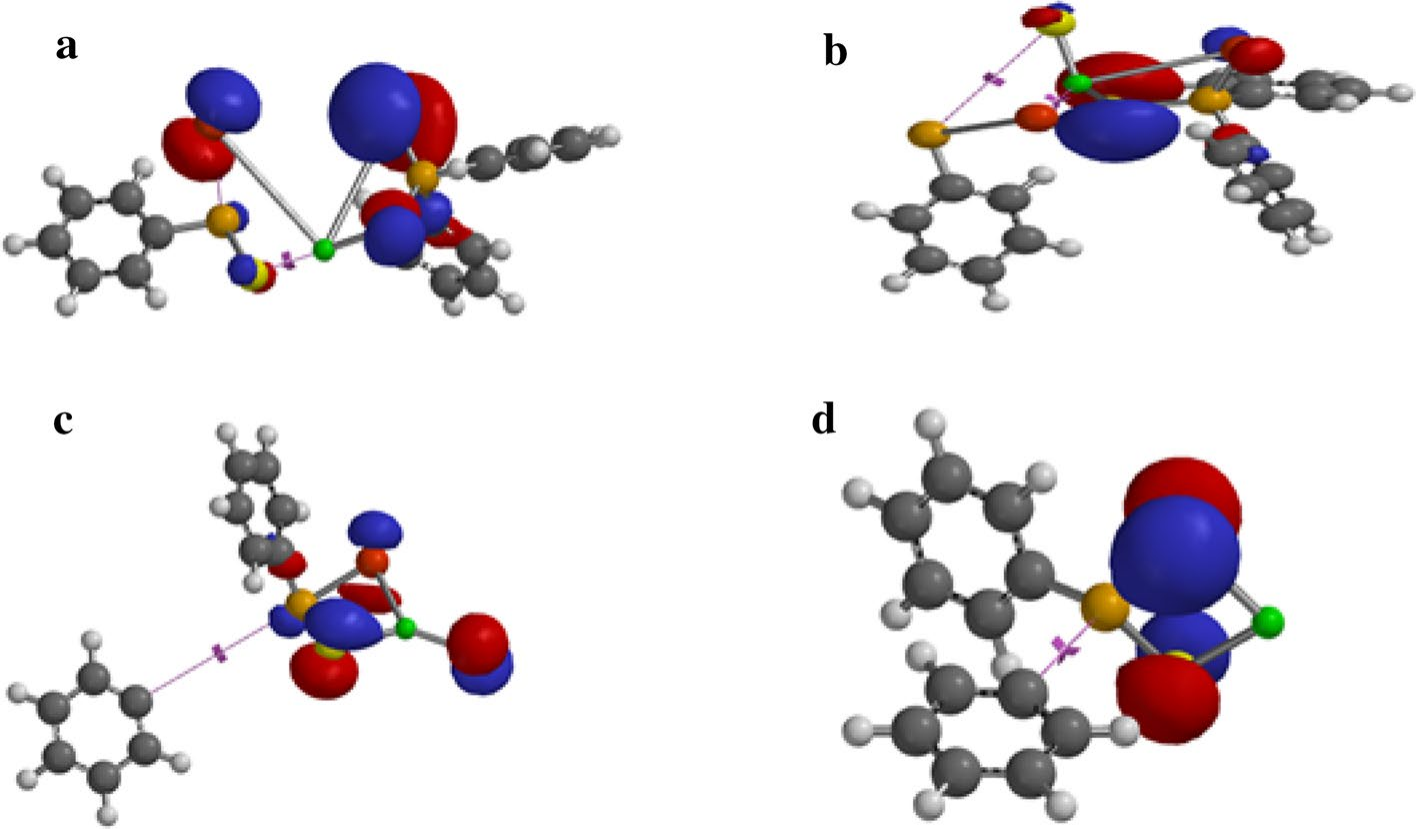
Singly occupied molecular orbitals for **a** (C_6_H_5_)_2_PSSeZnSe…SP(C_6_H_5_), **b** (C_6_H_5_)_2_PSSeZnS…SeP(C_6_H_5_), **c** C_6_H_5_…(C_6_H_5_)PSSeZnSe and **d** C_6_H_5_…(C_6_H_5_)PSSeZnS complexes. Isosurfaces ±0.032 a.u.

The SOMO in Figure 10a–d show an electron density distribution exclusively on the bridging ligand with no zinc contribution.

## Conclusion

In summary, we have studied the structural, electronic, kinetic and thermodynamic properties of Zn[(C_6_H_5_)_2_PSSe]_2_ precursor using the M06 method. Several possible pathways for the Zn[(C_6_H_5_)_2_PSSe]_2_ precursor and its subsequent decomposition emanating from (C_6_H_5_)PSSe–Pb–Se, (C_6_H_5_)PSSe–Pb–S, (C_6_H_5_)_2_P(Se)S–Pb and (C_6_H_5_)P(Se)S–Pb intermediates have been examined at T = 800K. According to our calculated results, the main conclusions are summarized as follows:

1. Pathways initiated via the formation of a ZnSe abstraction from the (C_6_H_5_)P(Se)S–Pb intermediate is competitive with the pathways that lead to the formation of ZnS.
2. Kinetically and thermodynamically the most favourable pathway involves the formation of ternary ZnSe_x_S_1–x_ on both the singlet and doublet potential energy surfaces.
3. The initial dissociation of phenyl radical to form the (C_6_H_5_)_2_PSSe–Zn–SeSP(C_6_H_5_) intermediate is kinetically only 3.47 kcal/mol lower than the [(C_6_H_5_)_2_PS]^−^ dissociation to form the (C_6_H_5_)_2_P(Se)S–Pb intermediate.
4. The isopropyl precursor appears to be more preferable to the phenyl precursor due to its high growth rate at their mutual deposition temperature.
5. The spin density map and single occupied molecular orbital analysis shows that it is mainly the coordinating Se- and S-donor atoms that provides the spin and electron contribution.
